# Supplementary material for: Taxonomic, functional and expression analysis of viral communities associated with marine sponges
Source: PeerJ. 2021 Feb 2;9:e10715. doi: 10.7717/peerj.10715 (PMC7863781; doi:10.7717/peerj.10715)
Supplement: Supplemental Information 2 [file peerj-09-10715-s002.docx]

## **Supplementary Information**

**Taxonomic, functional and expression analysis of viral communities associated with marine sponges.**

Mary THD Nguyen^1^, Bernd Wemheuer^1^, Patrick Laffy^2^, Nicole Webster^2,3^, Torsten Thomas^1^

^1^ Centre for Marine Science and Innovation & School of Biological, Earth and Environmental Sciences, The University of New South Wales, Sydney, NSW 2052, Australia

^2^ Australian Institute of Marine Science, Townsville 4810, Australia

^3^ Australian Centre for Ecogenomics, University of Queensland, Brisbane 4072, Australia


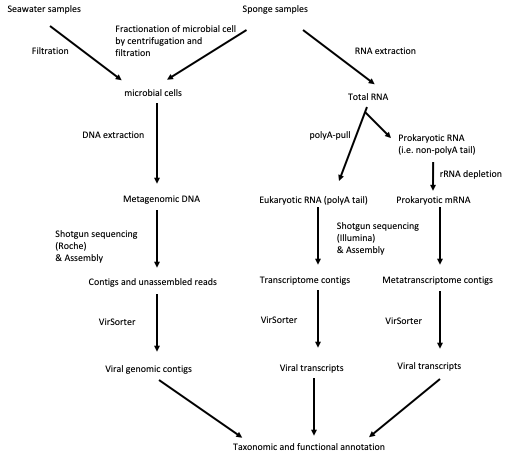


**Figure S1:** Flow chart of the sample processing and analysis performed.


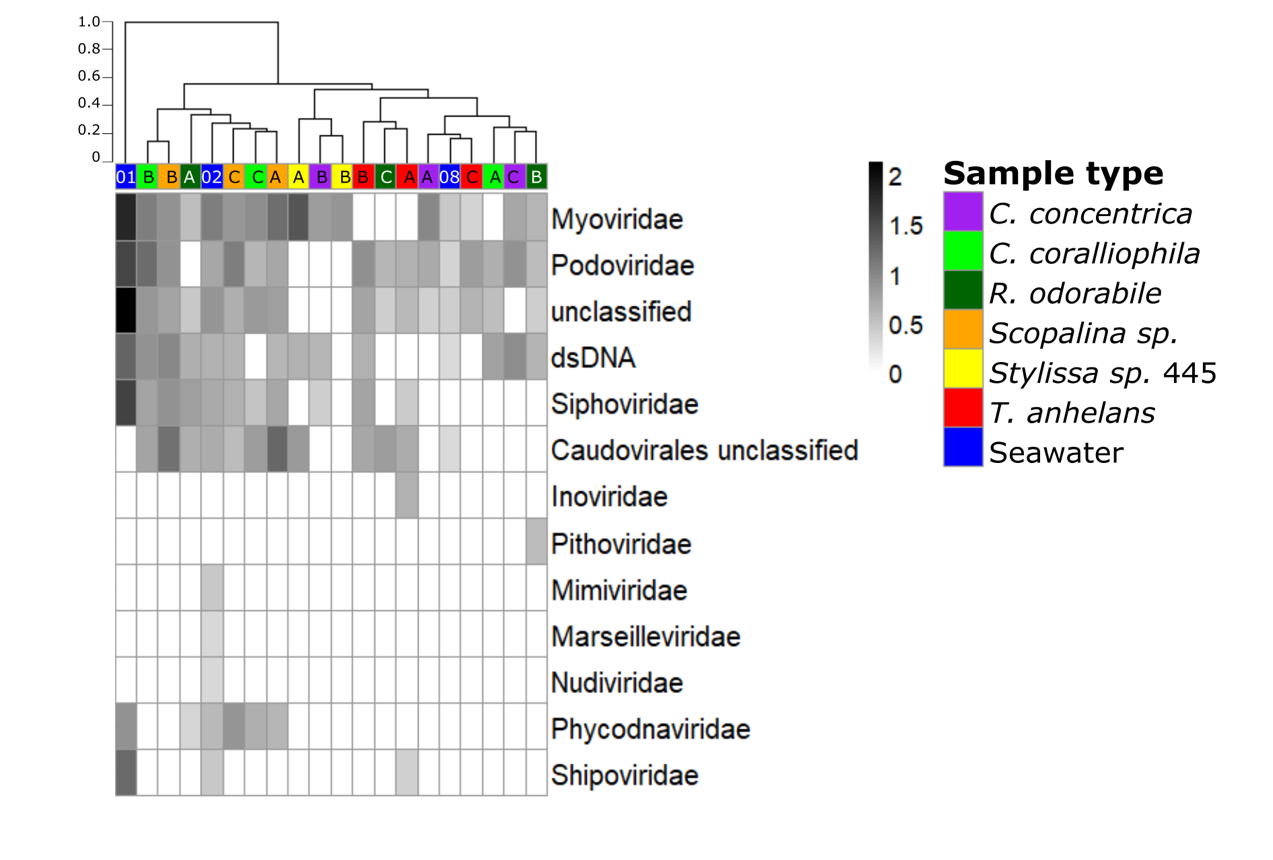


**Figure S2:** Viral communities based on metagenomic data and classified to the family-level (where applicable) are clustered based on Bray-Curtis dissimilarities of taxonomic profiles (tree scale indicates dissimilarity percentages). Values are normalised to viral genomes per prokaryotic genome transformed with fourth root. A, B and C indicate sample replicates for sponges and 01, 02 and 08 indicate replicates for seawater samples.


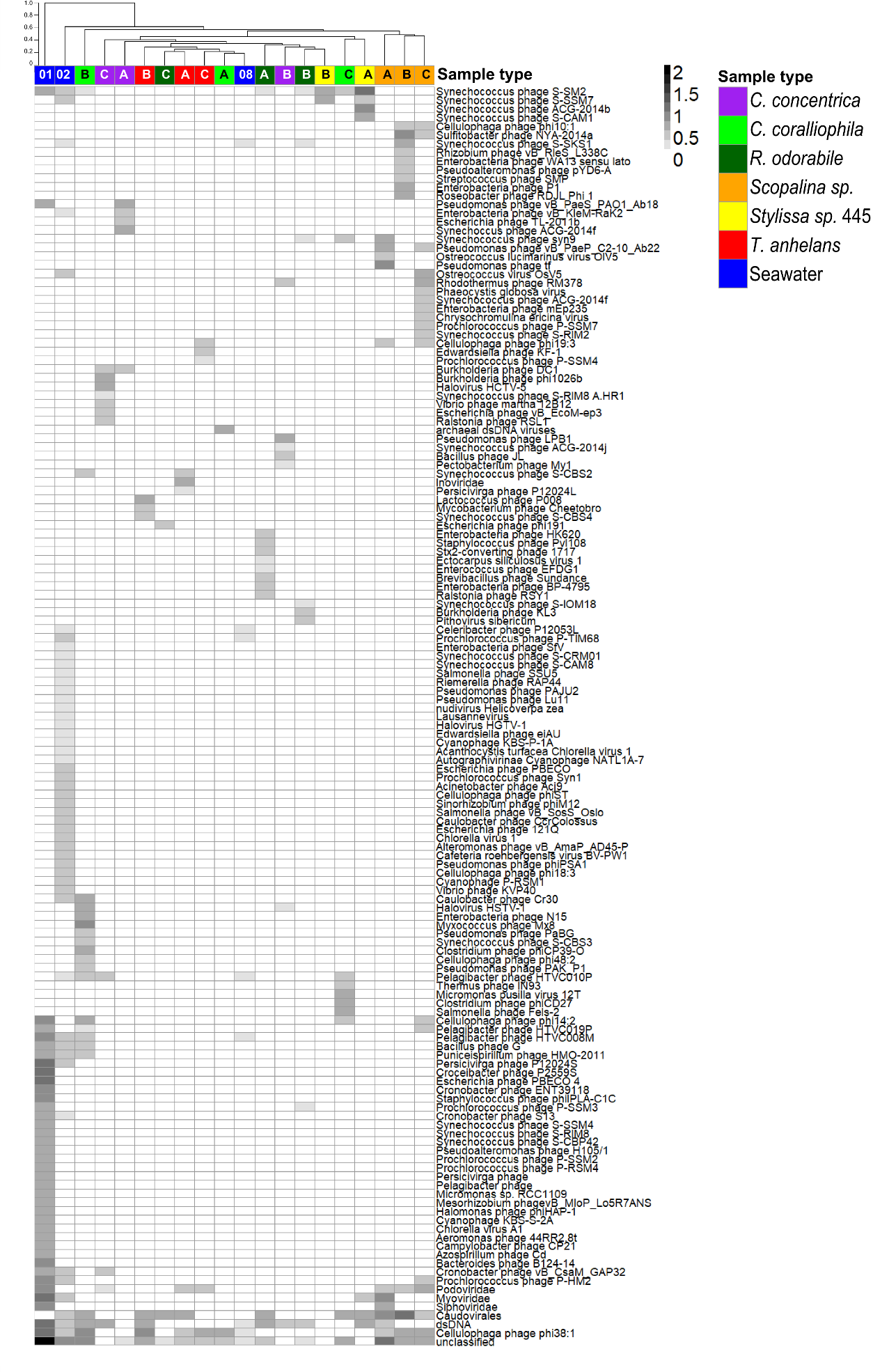


**Figure S3:** Viral metagenome communities in metagenomic data from sponges and seawater classified to the lowest possible classification level. Sample were clustered based on Bray-Curtis dissimilarity (tree scale indicates dissimilarity percentages). Values are normalised to viral genomes per prokaryotic genome transformed with fourth root. A, B and C indicate sample replicates and 01, 02 and 08 indicate replicates for seawater samples.


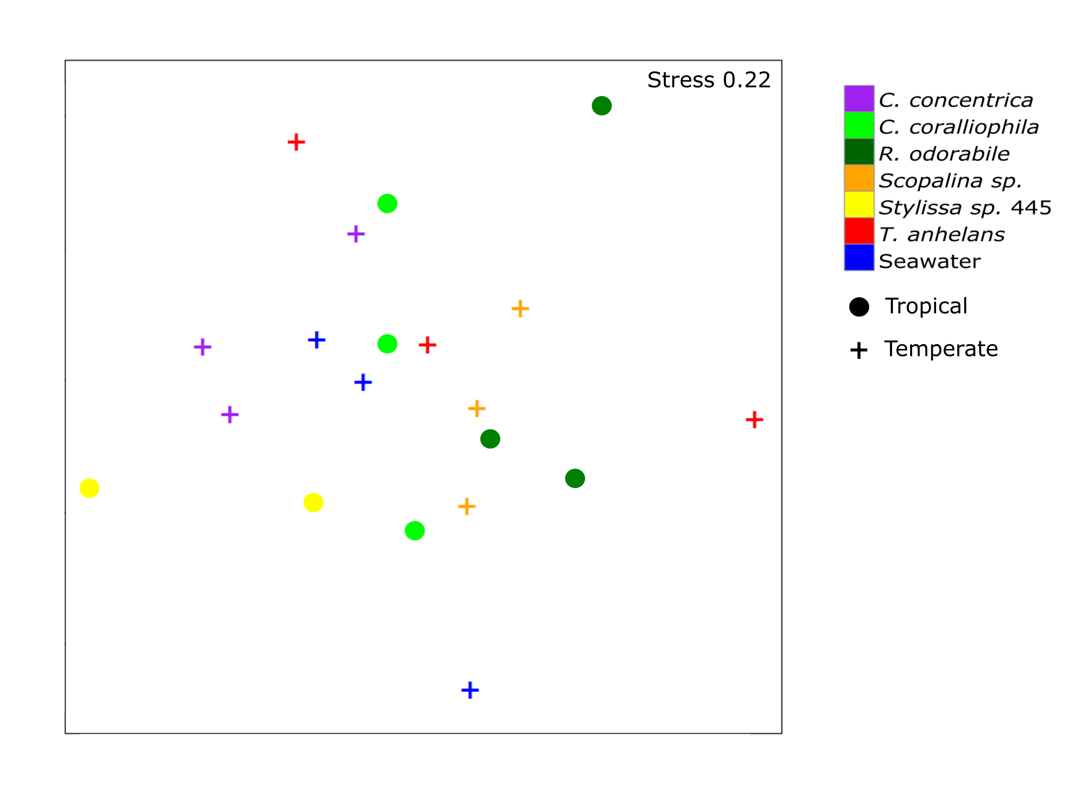


**Figure S4:** NMDS plot based on Bray-Curtis similarity of viral community composition on a family-level taxonomic assignment using metagenomic data from sponges and seawater. Host and environments are represented in different colours as shown in the legend. Sponges from tropical waters are indicated with a circle symbol and samples from temperate waters are indicated by a plus symbol.


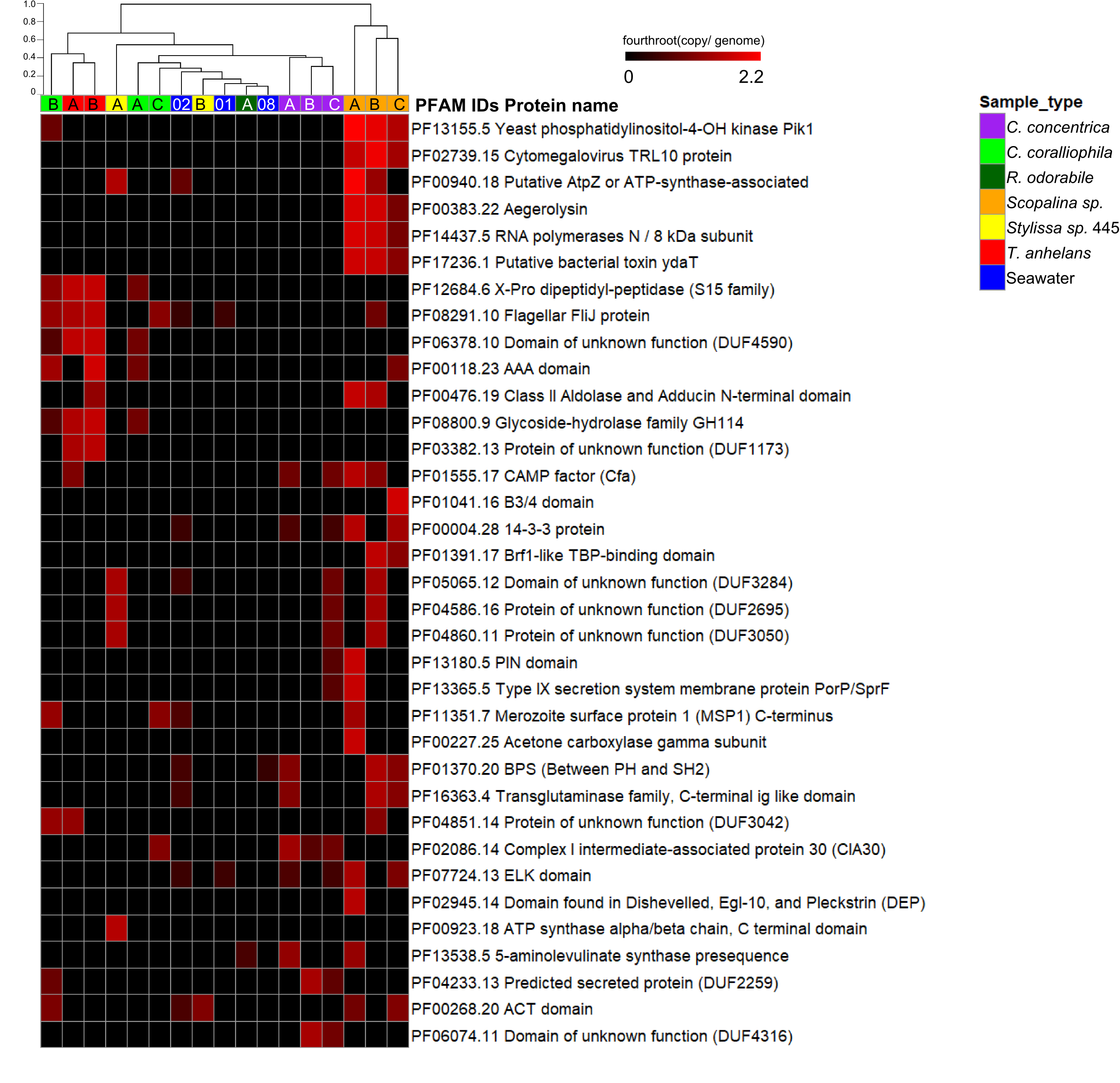


**Figure S5:** Top 35 most abundant proteins hits against the PFAM database for viruses in metagenomic data. Values are normalised to copies per genomes with fourth root transformation. Columns are clustered using the Bray-Curtis dissimilarities using the ‘average method’ and scale indicates percentages of dissimilarity. A, B and C indicate sponge replicates of sponges and 01, 02 and 08 indicate seawater replicates.


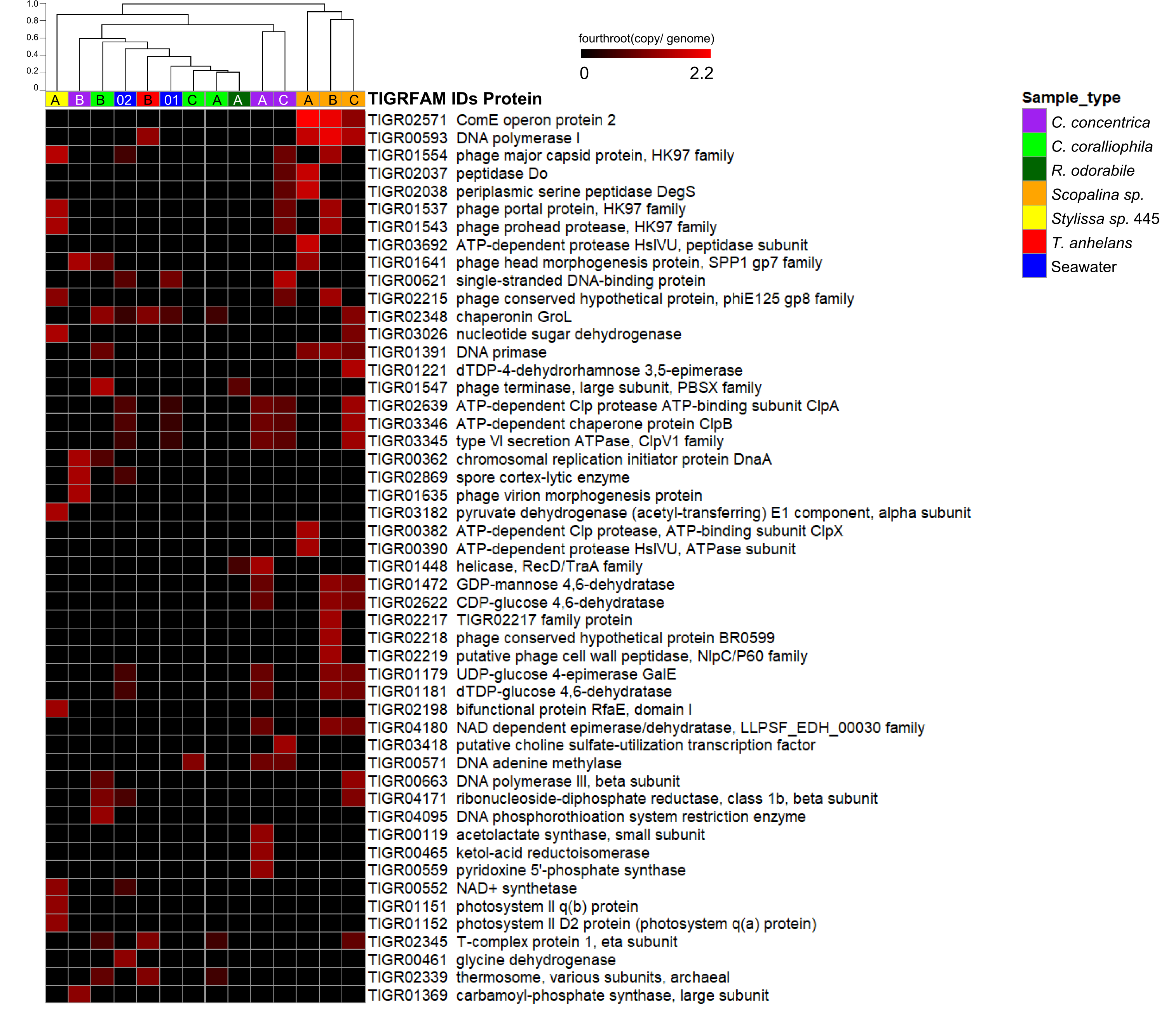


**Figure S6:** Top 50 most abundant proteins hits against the TIGRFAM database for viruses in metagenomic data. Values are normalised to copies per genomes with fourth root transformation. Columns are clustered using the Bray-Curtis dissimilarities using the ‘average method’ and scale indicates dissimilar percentages. A, B and C indicate sponge replicates and 01 and 02 indicate seawater replicates.


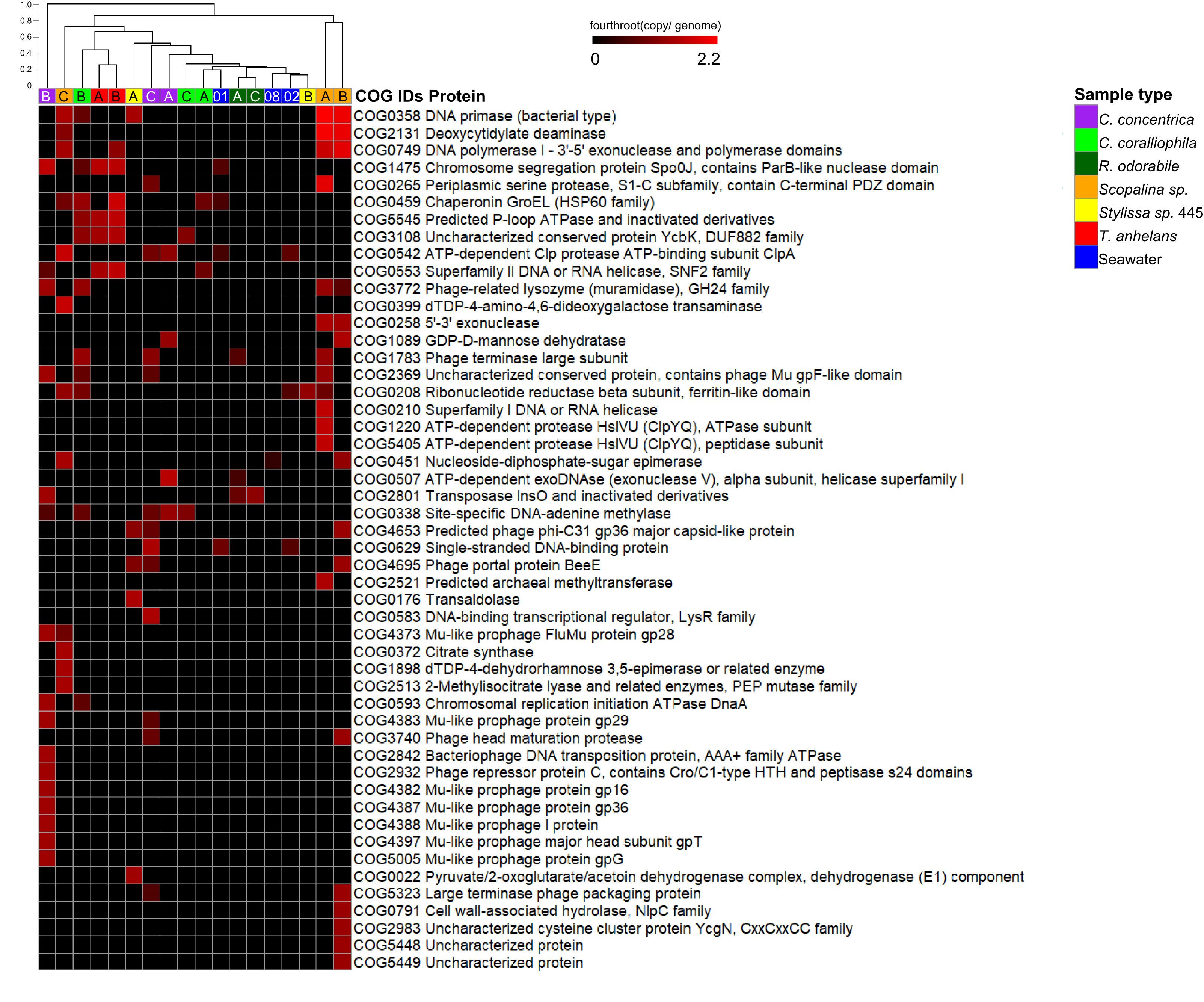


**Figure S7:** Top 50 most abundant proteins hits against the COG database for viruses in metagenomic data. Values are normalised to copies per genomes with fourth root transformation. Columns are clustered using the Bray-Curtis dissimilarities using the ‘average method’ and scale indicates dissimilar percentages. A, B and C indicate sponge replicates and 01, 02 and 08 indicate seawater replicates.


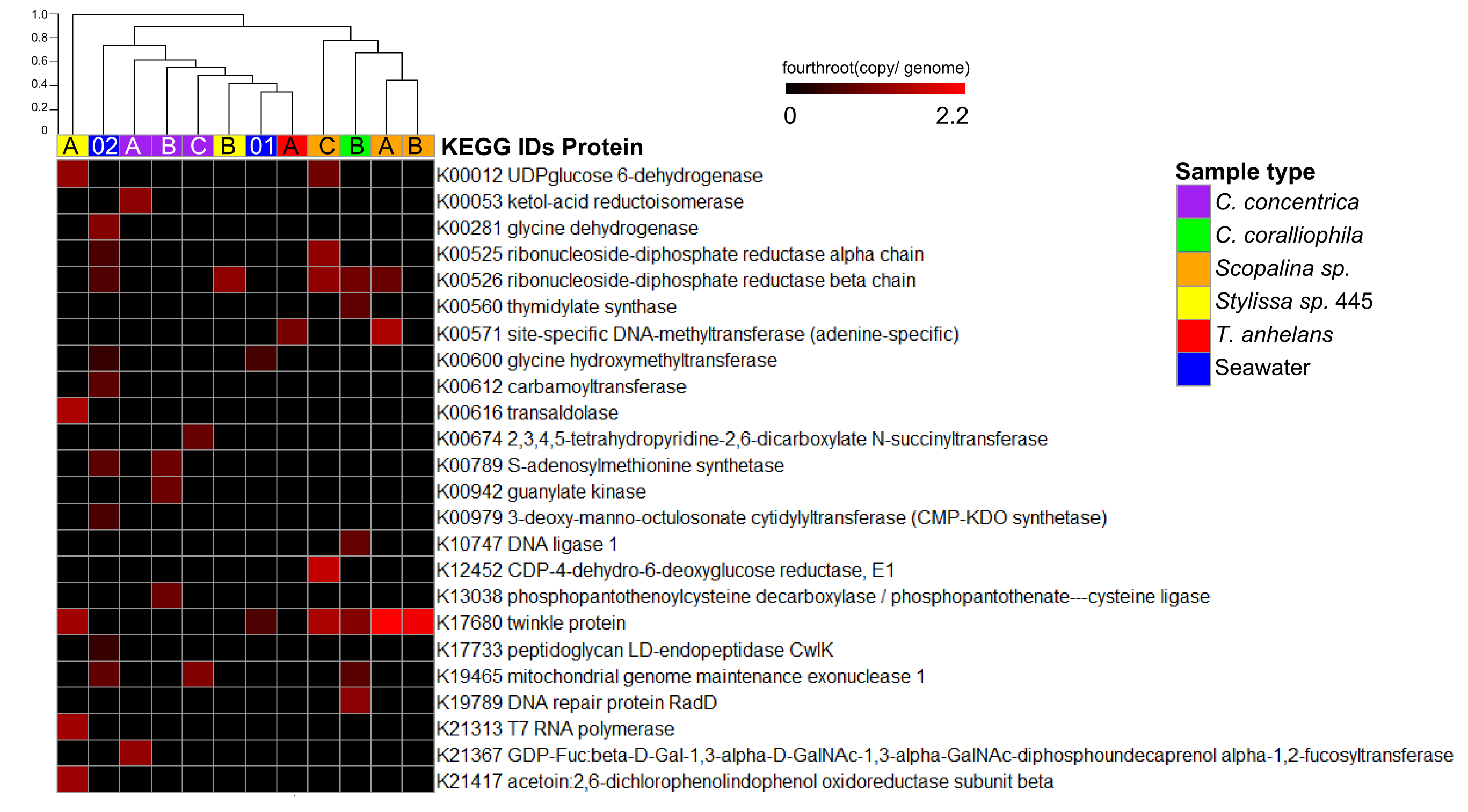


**Figure S8:** Proteins hits (e-value of 1E-10) against the KEGG database for viruses in metagenomic data. Values are normalised to copies per genomes with fourth root transformation. Columns are clustered using the Bray-Curtis dissimilarities using the ‘average method’ and scale indicates dissimilar percentages. A, B and C indicate sponge replicates and 01, 02 and 08 indicate seawater replicates.


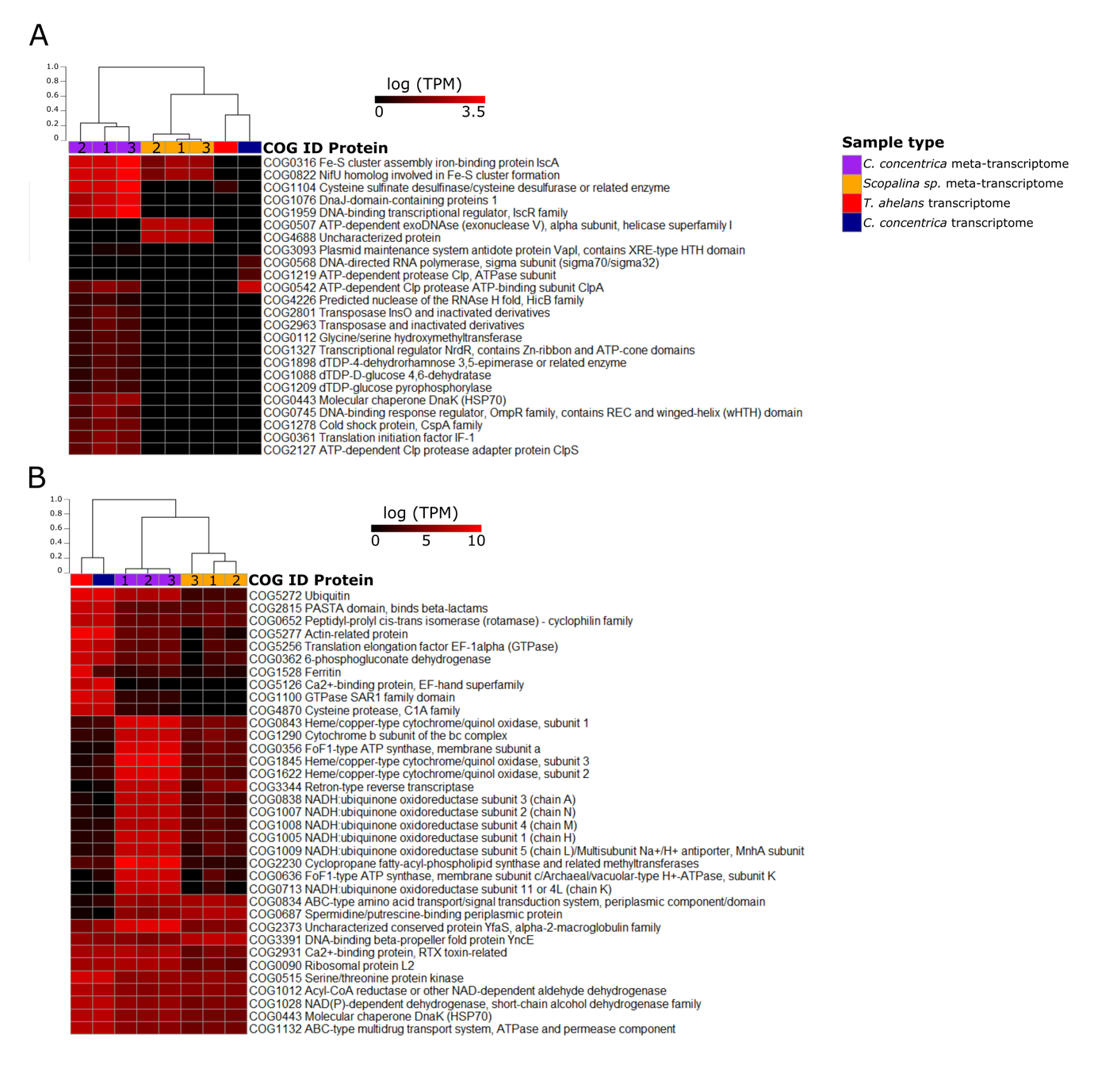


**Figure S9:** Top 35 most abundant proteins hits against the COG database for viral meta-transcriptomes and transcriptomes (A) and the whole meta/transcriptomes (B). Values are normalised to transcripts per million (TPM) with natural log transformation. Samples are clustered using the Bray-Curtis dissimilarities using the ‘average method’ and scale indicates dissimilar percentages. 1, 2 and 3 represent sample replicates. For transcriptome data, the three replicates were pooled together.


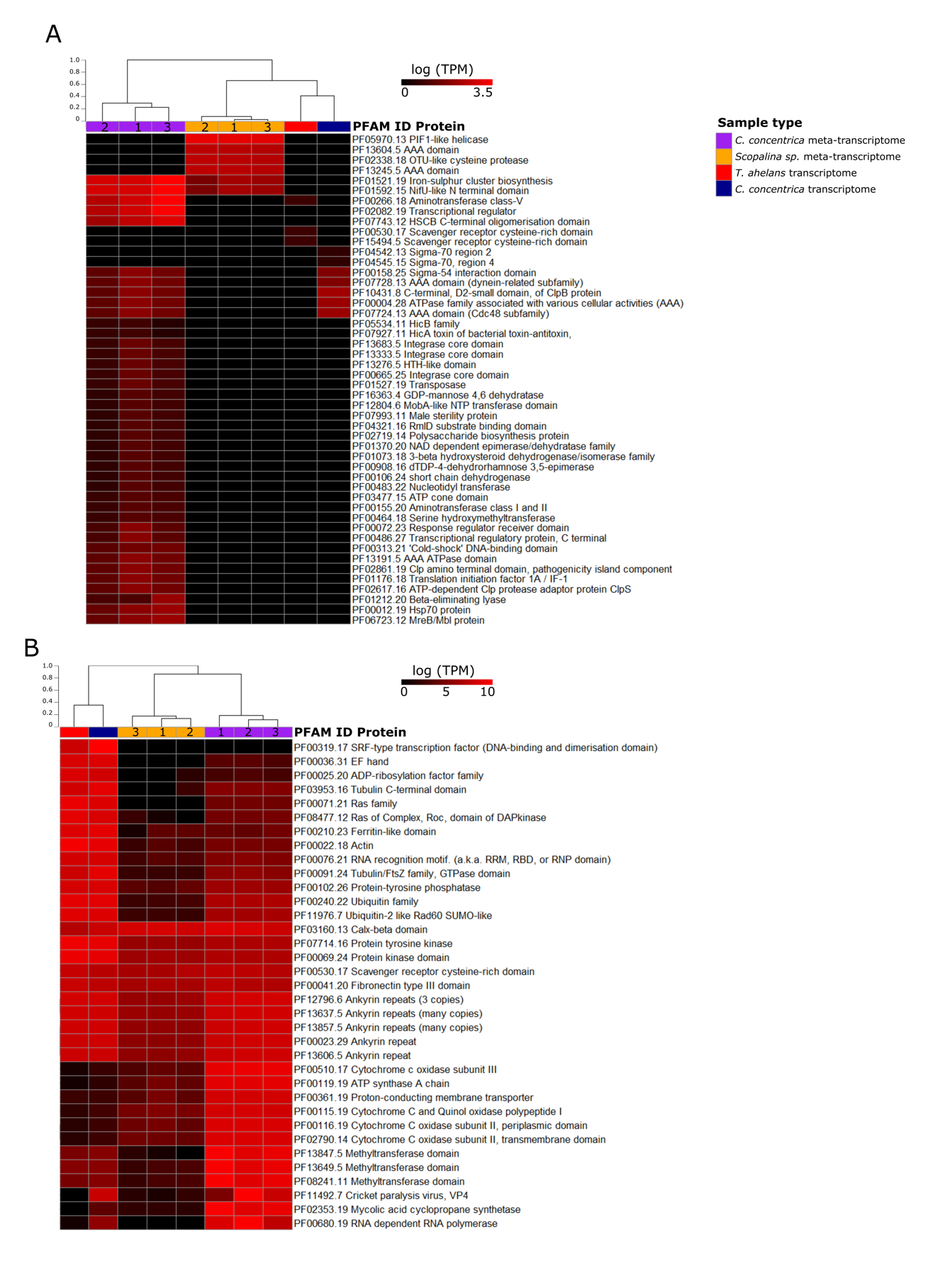


**Figure S10:** Top 35 most abundant proteins hits against the PFAM database for viral meta-transcriptomes and transcriptomes (A) and the whole meta/transcriptomes (B). Values are normalised to transcripts per million (TPM) with natural log transformation. Samples are clustered using the Bray-Curtis dissimilarities using the ‘average method’ and scale indicates dissimilar percentages. 1, 2 and 3 represent sample replicates. For transcriptome data, the three replicates were pooled together.


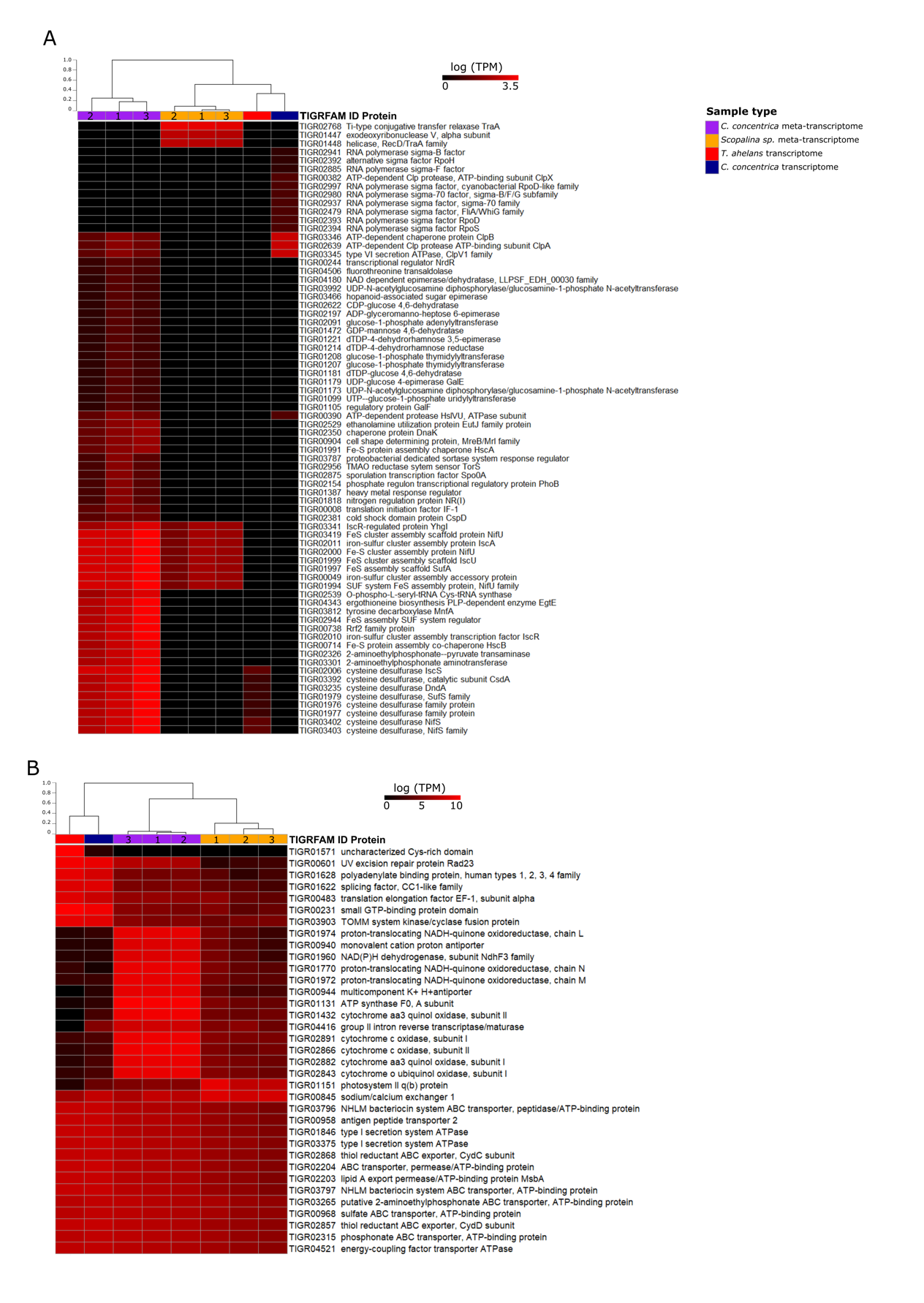


**Figure S11:** Top 35 most abundant proteins hits against the TIRGFAM database for viral meta-transcriptomes and transcriptomes (A) and the whole meta/transcriptomes (B). Values are normalised to transcripts per million (TPM) with natural log transformation. Samples are clustered using the Bray-Curtis dissimilarities using the ‘average method’ and scale indicates dissimilar percentages. 1, 2 and 3 represent sample replicates. For transcriptome data, the three replicates were pooled together.


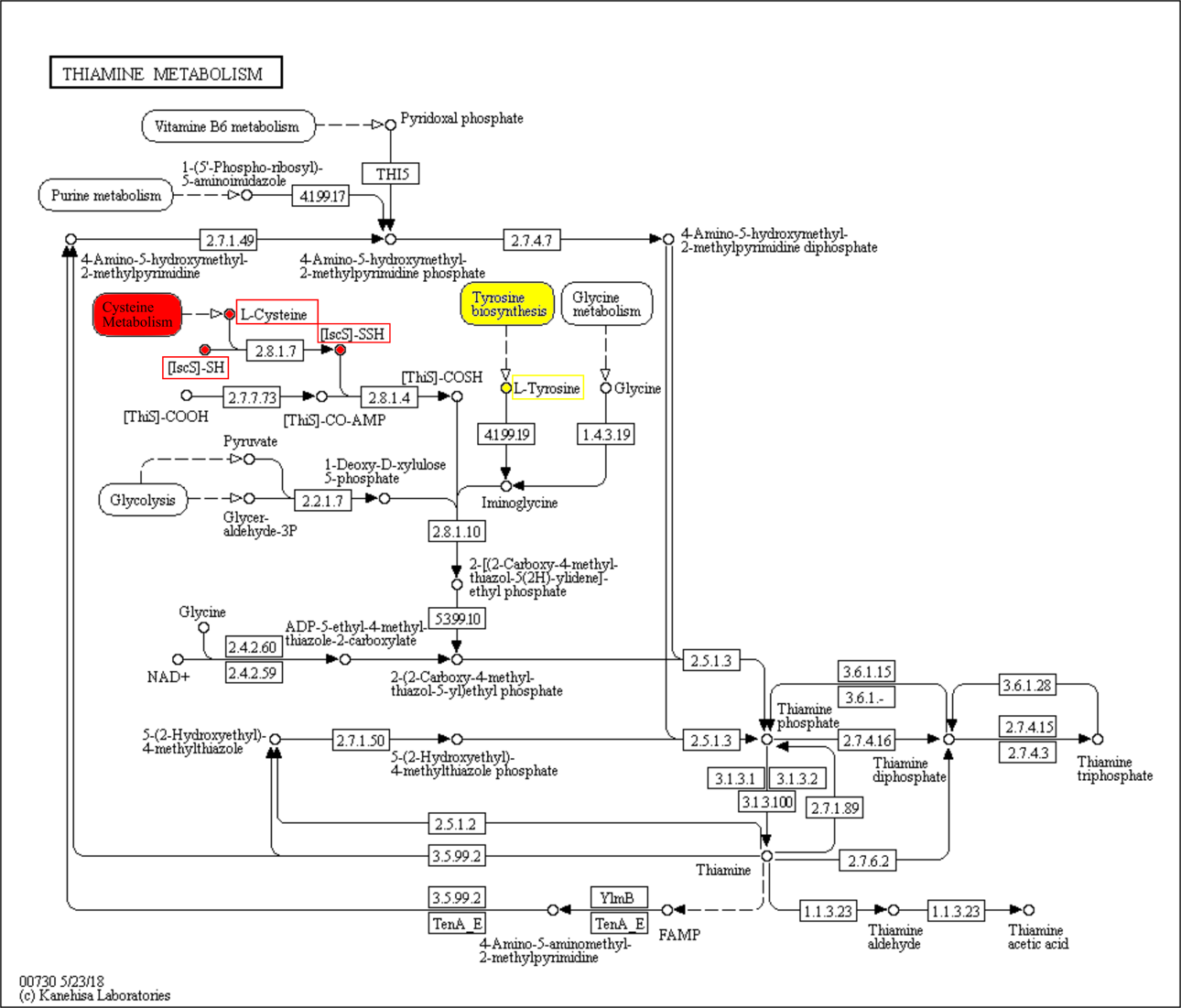

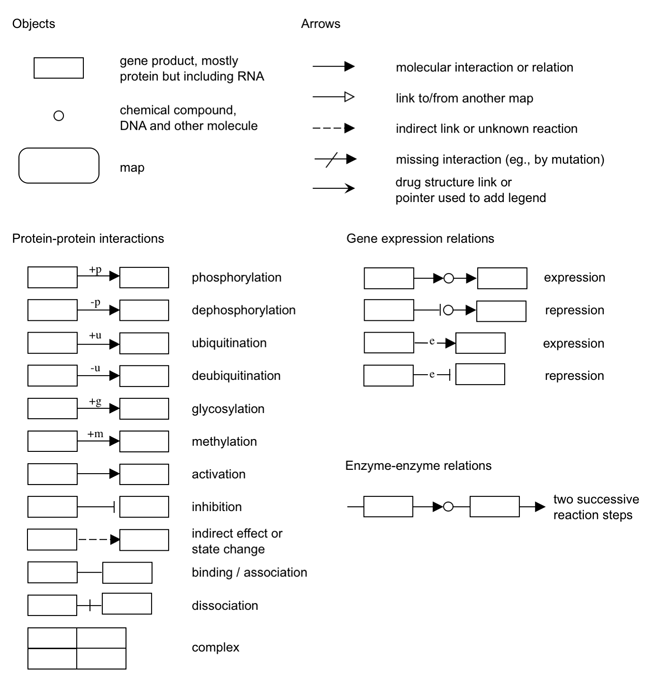


**Figure S12:** KEGG pathway map of thiamine metabolism and annotations. Tyrosine decarboxylase belongs to the tyrosine synthesis pathway which catalyses the production of L-tyrosine (highlighted in yellow). Cysteine desulfurase (IscS) is involved in transferring the sulfur from L-cysteine to ThiS (forming ThiS-COSH), which incorporates the sulfur into the thiazole ring of thiamine (highlighted in red).

**Table S1:** Shannon’s indices of viral assemblages associated with microbial cells in sponges and the Tukey multiple comparison of Shannon’s indices. Significant differences are taken at P ≤ 0.05 and are highlighted in bold.

| Shannon’s indices ± sd | | | | | | | | |
| --- | --- | --- | --- | --- | --- | --- | --- | --- |
| Sample | *C. concentrica* | | *C. coralliophila* | *R. odorabile* | *Scopalina sp.* | *Stylissa sp. 445* | *Seawater* | *T. anhelans* |
| Family-level | 0.78 ± 0.22 | | 1.34 ± 0.34 | 1.21 ± 0.46 | 1.54 ± 0.14 | 0.25 ± 0.36 | 1.6 ± 0.15 | 1.26 ± 0.50 |
| Species-level | 1.58 ± 0.28 | | 1.85 ± 0.85 | 1.60 ± 0.80 | 2.23 ± 0.41 | 0.96 ± 0.45 | 2.63 ± 0.69 | 1.67 ± 0.69 |
| Tukey multiple comparison of Shannon’s indices means (P-values of family-level, species-level) | | | | | | | | |
| *C. concentrica* | |  | 0.43, 0.99 | 0.69, 1.00 | 0.15, 0.81 | 0.61, 0.89 | 0.11, 0.36 | 0.60, 0.99 |
| *C. coralliophila* | |  |  | 0.99, 0.99 | 0.98, 0.99 | **0.04,** 0.63 | 0.95, 0.67 | 0.99, 0.99 |
| *R. odorabile* | |  |  |  | 0.88, 0.83 | 0.08, 0.88 | 0.78, 0.37 | 0.99, 0.99 |
| *Scopalina sp.* | |  |  |  |  | 0.01, 0.27 | 0.99, 0.97 | 0.93, 0.89 |
| *Stylissa sp. 445* | |  |  |  |  |  | **0.009,** 0.08 | 0.06, 0.82 |
| *Seawater* | |  |  |  |  |  |  | 0.85, 0.45 |

**Table S2:** PERMANOVA analysis of pair-wise comparisons of viral assemblages (at a family level) of microbial cells associated with sponges. Values indicated P-values and significant differences were taken at P ≤ 0.05.

| PERMANOVA P-values of viral family-level, species-level | | | | | | | |
| --- | --- | --- | --- | --- | --- | --- | --- |
| Samples | *C. concentrica* | *C. coralliophila* | *R. odorabile* | *Scopalina sp.* | *Stylissa sp. 445* | Seawater | *T. anhelans* |
| *C. concentrica* |  | 0.5, 0.1 | 0.1, 0.1 | 0.1, 01 | 0.7, 0.1 | 0.2, 0.2 | 0.1, 0.1 |
| *C. coralliophila* |  |  | 0.4, 0.2 | 0.7, 0.3 | 0.3, 0.1 | 0.8, 0.8 | 0.3, 0.5 |
| *R. odorabile* |  |  |  | 0.1, 0.1 | 0.1, 0.1 | 0.5, 0.1 | 0.2, 0.1 |
| *Scopalina sp.* |  |  |  |  | 0.2, 0.1 | 0.3, 0.1 | 0.1, 0.2 |
| *Stylissa sp. 445* |  |  |  |  |  | 0.2, 0.1 | 0.1, 0.1 |
| *Seawater* |  |  |  |  |  |  | 0.1, 0.1 |

**Table S3:** Proteins of the thiamine metabolism (TIGRFAM annotation) found to be expressed in the viral metatranscriptome of *C. concentrica*.

| TIGRFAM ID | Protein name |
| --- | --- |
| TIGR01976 | Am_tr_V_VC1184: cysteine desulfurase family protein |
| TIGR01977 | SufS: cysteine desulfurase |
| TIGR01979 | IscS: cysteine desulfurase IscS |
| TIGR02006 | DNA_S_dndA: cysteine desulfurase DndA |
| TIGR03235 | FeS_syn_CsdA: cysteine desulfurase |
| TIGR03392 | Catalytic subunit CsdA |
| TIGR03402 | FeS_nifS: cysteine desulfurase NifS |
| TIGR03403 | NifS_epsilon: cysteine desulfurase |
| TIGR03812 | Tyr_de_CO2_Arch: tyrosine decarboxylase MnfA |
| TIGR04343 | EgtE_PLP_lyase: ergothioneine biosynthesis PLP-dependent enzyme EgtE |

**Explanation of fasta ID for the file “Supplementary_data_viral_contigs_transcripts.fna”.**

Fasta header have the ID according to Fan et al. 2012 and Diez- Vives et al. 2017.

Contigs and unassembled long-read sequences from the cellular metagenomes are identified by “_contig” and “_F”, respectively, with sample type and replicates being indicated by the first part of the fasta header as per the abbreviations used by Fan et al. 2012.

Transcripts are indicated by the string “_TR” in the fasta header. Transcriptomic samples are indicated by the string “Euk”, while metatranscriptomic samples have the string “Meta”. Sponge species are indicated by “Cym”, “Sco” and “Ted” for *Cymbastela concentrica*, *Scopalina* sp. and *Tedania anhelens*, respectively.

Confidence categories from the VirSorter analysis are given after the string “cat_”.
